# Measuring attitudes towards mental health using social media: investigating stigma and trivialisation

**DOI:** 10.1007/s00127-018-1571-5

**Published:** 2018-08-01

**Authors:** Patrick Robinson, Daniel Turk, Sagar Jilka, Matteo Cella

**Affiliations:** 1 Department of Psychology, Institute of Psychiatry, Psychology and Neuroscience, King’s College London, De Crespigny Park, London, SE5 8AF UK; 2 South London and Maudsley NHS Foundation Trust, London, UK

**Keywords:** Stigma, Mental health, Social media, Twitter, Schizophrenia

## Abstract

**Background:**

There are numerous campaigns targeting mental health stigma. However, evaluating how effective these are in changing perceptions is complex. Social media may be used to assess stigma levels and highlight new trends. This study uses a social media platform, Twitter, to investigate stigmatising and trivialising attitudes across a range of mental and physical health conditions.

**Methods:**

Tweets (i.e. messages) associated with five mental and five physical health conditions were collected in ten 72-h windows over a 50-day period using automated software. A random selection of tweets per condition was considered for the analyses. Tweets were categorised according to their topic and presence of stigmatising and trivialising attitudes. Qualitative thematic analysis was performed on all stigmatising and trivialising tweets.

**Results:**

A total of 1,059,258 tweets were collected, and from this sample 1300 tweets per condition were randomly selected for analysis. Overall, mental health conditions were found to be more stigmatised (12.9%) and trivialised (14.3%) compared to physical conditions (8.1 and 6.8%, respectively). Amongst mental health conditions the most stigmatised condition was schizophrenia (41%) while the most trivialised was obsessive compulsive disorder (33%).

**Conclusions:**

Our findings show that mental health stigma is common on social media. Trivialisation is also common, suggesting that while society may be more open to discussing mental health problems, care should be taken to ensure this is done appropriately. This study further demonstrates the potential for social media to be used to measure the general public’s attitudes towards mental health conditions.

## Introduction

Attitudes towards mental health are still not on equal terms with those towards physical health. Stigma is recognised as a significant barrier for the early diagnosis and treatment of various mental health conditions [1]. The World Health Organisation has highlighted the significant role of stigma in influencing mental health prognosis and has identified its reduction as a key target in its 2013–2020 action plan [2].

Stigma is thought to be more prevalent in illnesses perceived to have uncertain or complex aetiology [3]. This may partially explain why stigma towards mental health conditions is much higher than it is to physical health problems [4]. Research by Rüsch et al., supports the link between poor knowledge and stigma, and showed that increased mental health literacy is associated with a reduction in stigmatising attitudes [5]. Furthermore, poor understanding of mental health conditions has been shown to be associated with public fear and the perception that people experiencing mental health problems are dangerous [6]. One of the most significant repercussions of stigma is its effect on help-seeking behaviour. People with mental health problems are less likely to seek help if they feel their condition is stigmatised [7, 8]. Stigmatising attitudes also isolate sufferers and make many societal roles, such as finding a job, harder [9].

Trivialisation is a minimising behaviour where an illness is conceptualised as being easier to acquire, suffer with, or treat. It may also be perceived as a form of stigma and has consequences. Recent research suggests that trivialisation can arise when diagnoses are introduced into common use without education on their meaning, for example using the term OCD to describe a personal preference regarding the arrangement of their belongings (e.g. “I’m OCD about tidying my room”) [10]. This may devalue the experience of those suffering from a mental health condition [11, 12]. Studies exploring the role of trivialisation in society suggest this may reinforce social inequality and this phenomena is more prevalent in mental health conditions [10, 13, 14].

Stigma and trivialisation are not equally distributed across different mental health conditions. Research has demonstrated that schizophrenia is one of the most negatively viewed conditions due to its misperception of danger and unpredictability. Similarly, studies show that eating disorders and depression appear to be stigmatised due to a perception of greater personal controllability [15, 16]. Stigma is also common in portrayals of physical conditions, and differs between individual conditions. A study of primary care attendees found that HIV was stigmatised significantly more than diabetes and hypertension [17]. Another study comparing stigma in AIDS and cancer found that AIDS was significantly more stigmatised than cancer due to societal attitudes towards homosexuality and religion [18].

Previous studies have assessed stigma using media portrayals of mental illness. They were found to include disproportionately high levels of stigmatising references to dangerousness and violence, but these studies are limited by low response rates, a reliance on surveys and traditional media anchoring effects [19–22]. These issues have prompted the use of alternative approaches and attention has turned to social media [23].

Two recent studies have used Twitter to assess stigmatising attitudes towards mental and physical illness. Reavley and Pilkington found tweets containing #schizophrenia were significantly more stigmatised than those containing #depression. Joseph et al. showed that schizophrenia was more stigmatised than diabetes on social media [24, 25]. These studies were limited by short sampling periods and by sampling tweets containing only two specific hashtags. It is likely that searching larger phrases and key words may return views people express in conversations and this may be more representative of public opinion. A further limitation of both these studies is that only one mental and one physical health condition were compared, diminishing the opportunity to observe trends both within and between mental and physical health problems.

In this study, we assess the prevalence of both stigmatising and trivialising attitudes in a great number of messages from a large social media platform (i.e. Twitter). We are aiming to contrast attitudes within different mental health conditions, as well as between different mental and physical health conditions. We also conduct a qualitative analysis on stigmatising and trivialising tweets to highlight specific content and trends.

## Methods

### Search terms generation

We compiled a comprehensive list (i.e. 50) of common physical and mental health conditions of varying aetiology, mode of transmission, time course, reversibility and system affected. Each term was searched using ‘Topsy’ (a real-time analytic tool for Twitter to collect tweets) over a 30-day period to ensure that each condition returned a sufficiently large number of tweets. Due to the way that our tweet-aggregation software worked, we were only able to search for conditions with single-word names or acronyms. Of the terms considered we selected the five mental and five physical health conditions with the most tweets with names that would allow us to collect tweets about them (i.e. these conditions all returned over 50,000 tweets and had single word/acronym search terms). These included schizophrenia, obsessive compulsive disorder, depression, autism, eating disorders, asthma, diabetes, HIV/AIDS, cancer and epilepsy. Search terms (see “Appendix 1”) were searched in the noun and adjectival forms of the word where possible, as described in Joseph et al. [25].

### Data collection

Data were collected in ten 72-h-long blocks which were equally distributed throughout the 50 days between 9th December 2015 and 27th January 2016. We used the Twitter Archiver add-on for Google Sheets together with Twitter Advanced Search to automatically collect tweets containing the above terms every 15 minutes [26]. This includes references to the target conditions with or without hashtags in the tweet, ensuring a more representative sample of tweets compared to previous studies [24, 25]. By sampling every 15 min from Twitter’s application programming interface (API), we aimed to capture more than the 40% of the total tweets produced as calculated by Morstatter [27]. From this sample, we then selected a random subset of 1300 tweets per conditions to be used in the analysis. Random selection was performed with STATA ver.14.

### Tweet rating

Of the tweets retrieved, some were excluded based on the following criteria: (a) part of or all the text was written in a language other than English; (b) the target word was used in a context other than that of the target medical condition (e.g. economic depression); (c) information in the tweet was limited (e.g. tweets consisting mainly of hashtags); (d) tweet content refers to the target condition in animals; (e) tweets containing the target word and pictures only. All remaining tweets were considered for analysis.

The rating criteria for tweets were based on previous literature [24, 25] and refined using an iterative rating exercise on a set of 1000 random tweets between two authors (PR and DT). Raters initially created a set of definitions by examining recurring themes across tweets, then tested these on a different set of tweets and refined them in consultation with a third author (MC) until the inter-rater reliability exceeded 0.8. Tweets were also given a mutually exclusive general theme based on their purpose to identify trends in the type of tweet. We added all definitions to a coding manual (see “Appendix 3”).

### Quantitative analysis

Descriptive statistics (i.e. frequency counts) were initially used to estimate the prevalence of stigmatising and trivialising tweets per condition. A Chi-square test was used to test the difference in the proportion of stigmatising attitudes between categories. All analyses were conducted using SPSS version 22.

### Qualitative analysis

Qualitative analysis, using a content analysis framework, was performed to add further detail on how stigmatisation and trivialisation occur on social media [28]. All tweets were coded and labelled independently by two authors (PR and DT). Tweets coded as stigmatising or trivialising were further coded in sub-categories in relation to language and emotional valence.

## Results

**Fig. 1:**
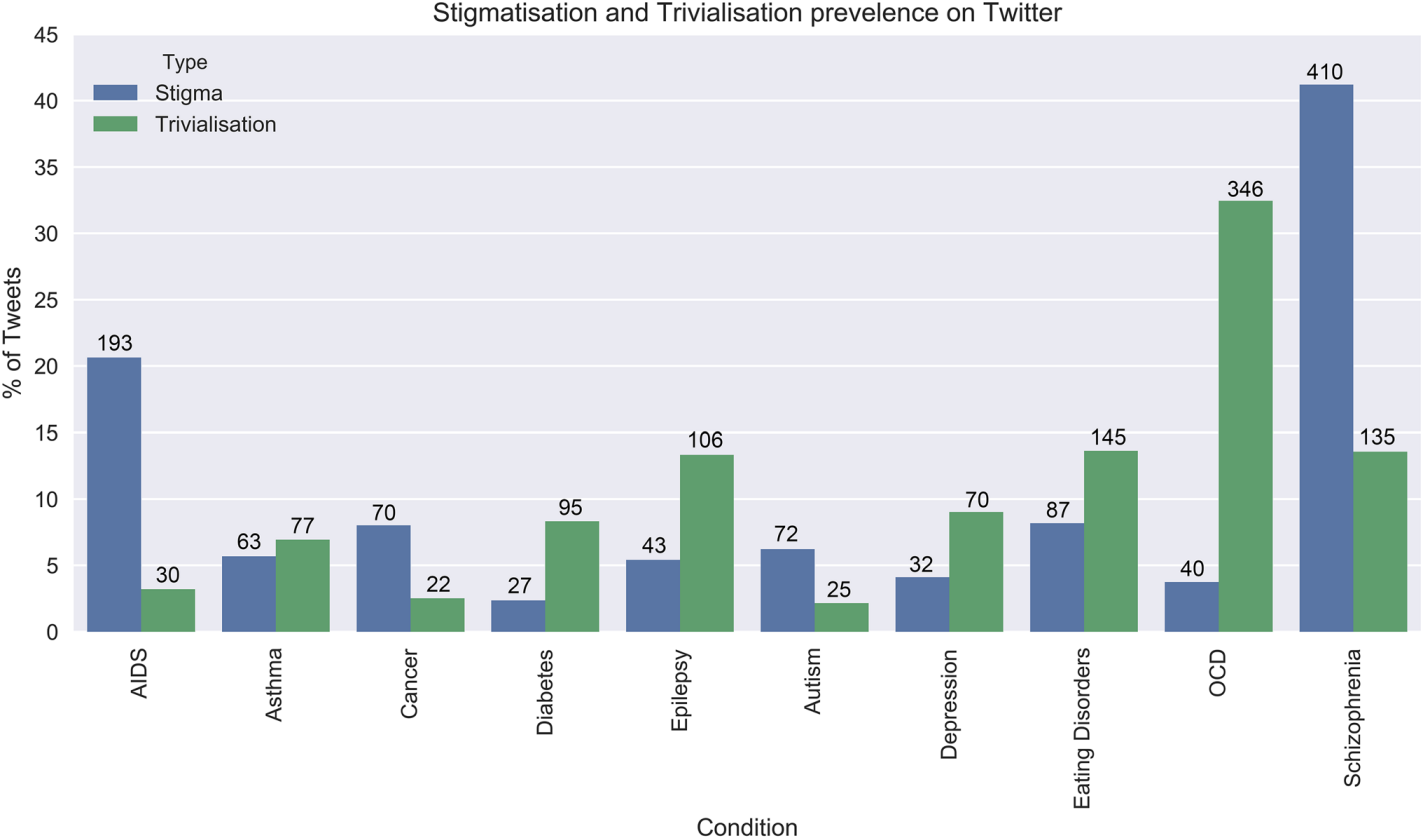
Stigma (blue) and trivialisation (green) across all conditions (*x*-axis) as a percentage of total tweets (calculated as number of tweets/included tweets × 100) on the *y*-axis, with the number of tweets for each condition recorded above each bar

**Fig. 2:**
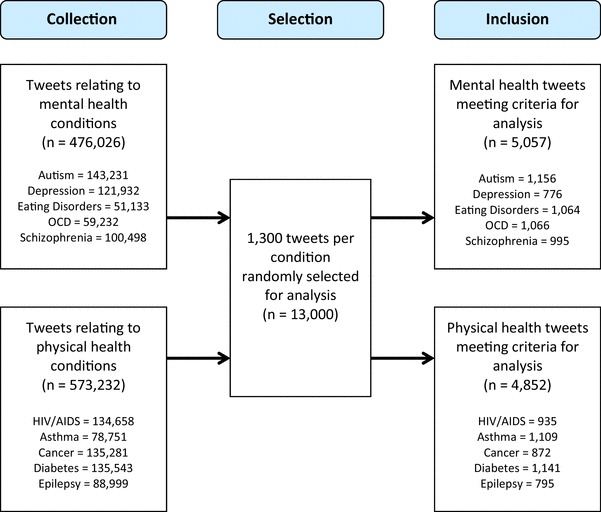
Number of tweets collected and included by stigma and trivialisation across all physical and mental health conditions

The prevalence of stigmatising and trivialising tweets for each condition is presented in Fig. 1. The total number of tweets collected is detailed by condition in Fig. 2. Of the 1300 tweets per condition, there was some variation in the number of tweets that we could consider based on our exclusion criteria.

The average stigma prevalence for the five physical health conditions considered was 8.1% whilst for the five mental health conditions was 12.9%. Analysis comparing the prevalence between categories showed that mental health conditions were 1.54 times more likely to be stigmatised, *χ*^2^(1, *N* = 9909) = 53.95, *p* < .001. The prevalence of trivialisation in physical health conditions was 6.8% while in mental health conditions was 14.3%. Trivialisation was 2.10 times more prevalent in mental illness than in physical illness, *χ*^2^(1, *N* = 9909) = 146.40, *p* < .001, (see Fig. 1). Both the most stigmatised (schizophrenia) and trivialised (OCD) conditions were mental health conditions.

**Fig. 3:**
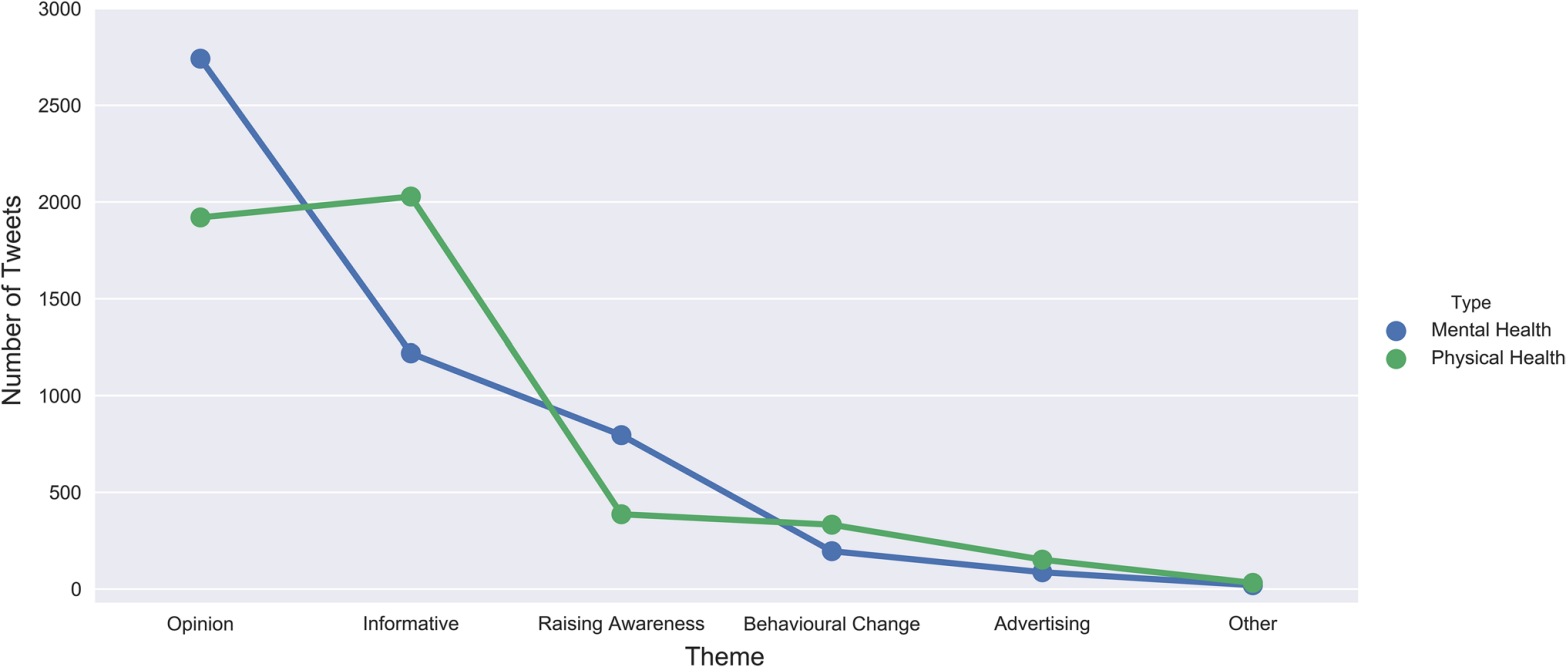
Number of tweets by theme over physical health (green) and mental health (blue) conditions

A Chi-square test performed to examine the proportion tweets by theme (see Fig. 3) showed that stigmatising tweets were more likely to occur in “Opinion” tweets, *χ*^2^ (5, *N* = 9911) = 1234.33, *p* < .001.

### Qualitative analysis

Trivialisation and stigma were present across all conditions, but differed slightly in their presentation within and between physical and mental health conditions. The emerging categories across the trivialising tweets were: (A) trivialising acquisition; (B) trivialising suffering; (C) minimising recovery difficulty (‘snap out of it’ in a previous study); (D) mockery (with a negative humour element) and (E) glamourising, or using the illness as a compliment [24]. Within the mockery theme we noticed a higher prevalence of tweets associated with benefit in mental health conditions.

For stigmatising tweets we identified the following sub-themes: (A) negative descriptor (using the illness to describe something in a negative light); (B) wishing illness upon someone (wishing harm upon someone by way of contracting the target condition); (C) negative characteristics (associating the illness with undesirable attributes); (D) joking (demeaning the target condition by joking about it) and (E) stereotyping (associating the illness with grossly inaccurate stereotypes). Examples of tweets by category can be found in “Appendix 2”.

## Discussion

The main aim of this study was to quantify the prevalence of stigmatising and trivialising attitudes across physical and mental health conditions on social media. The results show that mental health conditions were subject to more stigmatising and trivialising attitudes than physical health, but there was a large variation in prevalence between conditions.

Our findings that schizophrenia and HIV were the most stigmatised conditions is consistent with much of the previous research on this topic using different methods [15, 17, 25]. Both conditions share a perception of being dangerous—HIV/AIDS as a highly infectious and poorly understood disease, and schizophrenia perceived as unpredictable and difficult to control. Many tweets used ‘psychotic’ as an insult, and this is likely due to a deeply entrenched culture of negativity surrounding schizophrenia reinforced by media stereotypes.

The observed trends of stigma generally reflect those seen within the literature but the prevalence appears to be greater compared to previous studies [24]. Whilst this could suggest that our definition of stigma is more sensitive, this study was the first to consider whole tweets rather than just hashtags, which may have uncovered stigmatising attitudes that had not previously been assessed. Other strengths of this study, compared to previous research in this field, were the large population size, comprehensive tweet collection and the random sampling method, which ensured a representative sample of tweets for each target condition. By using automated software, we could retrieve a larger number of tweets compared to all previous studies in this area, and we also considered a wider range of target conditions.

Our methodology also allowed us to build on previous research and use qualitative analysis to compare tweet themes between mental and physical illnesses. This showed that mental health conditions were more likely to be discussed through opinion rather than factual discourse and tweets in the opinion theme were more likely to be stigmatising, while physical health conditions were more likely to be discussed via informative tweets (see Fig. 3). We think this is notable as it reaffirms the idea that stigma is often driven by (misinformed) opinion, and concerted campaigns to increase the informative content in discussions of mental illness on Twitter could form the basis for future stigma-reduction strategies.

Although this study improves on previous research, there were still several limitations. The rating process meant there was an inherent degree of subjectivity due to differences in the perceived context and emotional tone of some tweets, and the inability to follow links and embedded pictures. This was made particularly evident by words that had dual meanings (e.g. cancer, depression). There was also a degree of selection bias as stigmatising and trivialising tweets were more likely to be lacking in context and/or grammatical correctness, rendering them less likely to be considered for analysis. We minimised the impact of these issues through our robust rating criteria and repeated inter-rater reliability testing. A binary rating system was chosen as it allowed us to rate a larger number of tweets but it may have obscured important differences in the mechanisms by which conditions are stigmatised and trivialised. Due to the information available via the API, we were unable to control for potential confounding variables such as demographic characteristics.

The limitations of this study provide several opportunities for refinements in any future studies. These include a non-binary approach to rating stigma tweets, evaluating re-tweets (perhaps as a proxy of endorsement) and analysis of the profile that generate the tweet (e.g. activity and number of followers).

It can be difficult to infer context from tweets. Systematic incorrect inference can lead to either overestimation or underestimation of stigma and trivialisation prevalence. Studies in this area should consider carefully how tweets are rated. The difficulty can be illustrated by the following two examples. The tweet: ‘I can; seizure salad’ contains little context and it is not possible to determine whether the user is trivialising the condition, or has simply misspelt Caesar. While the adjective depressed is often used to infer low mood and the sufferance of clinical depression, some tweets were ambiguous (e.g. “I have a deep love for depressed comedians”) and therefore had to be excluded. From 140 characters or less it can be difficult to unambiguously infer meaning.

We have shown that stigma and trivialisation are highly prevalent on social media and that, as an ever-greater proportion of social interaction takes place online, proactive campaigns should consider assessing and addressing both on social media platforms. We believe our study can contribute to develop the knowledge necessary to build computer algorithms capable of detecting stigma on social media and give us the opportunity to target anti-stigma campaigns to those who may benefit from it most. This is the same logic used by commercial advertising where product advertising is targeted to potential consumer preference inferred by the way they use social media. Targeting anti-stigma campaigns to individuals’ profile may prove useful to educate and change attitudes towards mental health conditions.

## Appendix 1

Description of the Twitter search terms used in the noun and adjectival forms for all physical and mental health conditions, as described in Joseph et al. [25].

**Table:**

Condition	Search terms used
Physical health	
AIDS	HIV OR AIDS
Asthma	Asthma OR asthmatic
Cancer	Cancer OR cancerous
Diabetes	Diabetes OR diabetic
Epilepsy	Epilepsy OR epileptic OR seizure
Mental health	
Autism	Autism OR autistic OR asperger OR Asperger’s
Depression	Depression OR depressive OR depressed
Eating disorders	Anorexia OR anorexic OR bulimia OR bulimic OR OSFED OR EDNOS
OCD	OCD
Schizophrenia	Schizophrenia OR schizophrenic OR psychosis OR psychotic

## Appendix 2

Examples of stigma and trivialisation tweets by themes. These include; (A) Negative descriptor (using the illness to describe something in a negative light); (B) Wishing illness upon someone (wishing harm upon someone by way of contracting the target condition); (C) Negative characteristics (associating the illness with undesirable attributes); (D) Joking (demeaning the target condition by joking about it) and (E) Stereotyping (associating the illness with grossly inaccurate stereotypes).

**Table:**

Theme	Trivialisation examples	Stigma examples
(A) Negative descriptor	@*** the way he spelled my name gave me aids	Blacktown parking is aids
	RT@***: I think I’m having an asthma attack! Because you take my breath away!;) #CheesyPickUps	Has anyone seen the new torn & jerry orbugz bunny? S**t is actual cancer you have the worst slang
	A driver amused himself at the red lights by playing his ukulele. And now you have type two diabetes from reading that piece of saccharine	You look like aids
(B) Wishing illness upon someone	RT@*** If you have asthma just breathe lol	Get cancer faggot we are all your masters now
	RT @*** How you anorexic smh just eat RT@***: ~***~-his laugh will cure depression and cancer	After what I saw you doing with your nympho friend tonight looking for a f**k I’m done. Go ahead and be like her and get AIDS
(C) Negative characteristics	RT@***; I have so much OCD when it comes to the notifications on my phone	I like a pretty boy black man, not them dirty ass nasty AIDS carrying ashy elbow & lips havin Chief Keef mofos
	RT@***: *** said’ I CAN KILL ANYONE & NOTHING ’LL HAPPEN! Illiteracy is worse than Ebola, cancer, HIV!*** disgrace RT@***: Twitter is turning into Facebook and it’s giving me cancer	Pakistan Is The World’s Only Schizophrenic Nuclear State Brad Sherman
(D) Joking	RT@***: Just got a flu shot feeling autistic	Phone sex is dangerous, it may lead to hearing aids
	Based on what I saw Victoria has the Secret for anorexia... She can keep that	I just farted and it sounded like a kid with asthma trying to play the trumpet
	RT@***: if steve harvey was bulimic he’d be heave starvey	Depression runs in my family. We have blue genes
(E) Stereotyping	I wish anorexia was something I could catch, I could really use it rn	OK TIME FOR A SING SONG X IF YOUR OCD AND YOU KNOW IT WASH YOUR HANDS
	You n***z bout Sweet as hell every time y’all talk and open y’all mouth feel like a n***a gonna catch diabetes	Im your stereotypical nerd kid I have asthma and I wear glasses
	RT@***: ur psychotic, but I’m into ur flavor of psychotic	My mom didn’t raise a fool. A crazy psychotic b***h... But not a fool

## Appendix 3

**Table:**

Tweet theme	Description of tweets	Example
Raising awareness	Aim to raise awareness, address misconceptions or actively combat stigma about target condition	Please could you help raise awareness of pure ocd by retweeting https://t.co/VQcXGkiqjS
Advertising	Encourage purchase, or publicise a job opportunity (not including tweets with an expressed aim of raising awareness)	Nature made diabetes health Pack—provide the nutrients that you may be lacking in https://t.co/L9vslMcRZl Prediabetes
Informative	Propagate or solicit information without personal opinion or elements from other coding categories	Omega-3 deficit causes depression #health https://t.co/r5yeakFtUQhttps://t.co/Z5YAZtjy9rhttps://t.co/PC0gb5XQLi
Opinion	Convey or elicit emotion through the expression of personal opinion or felt experience; OR Pass on personal information	Seriously though, F*** you cancer! Stop stealing people away from us
Behavioural change	Empower or educate individuals to enable avoidance of, or coping and caring strategies for, the target illness. May encourage proactive behavioural change without mention of further purchase	Do not forget your inhaler when it’s cold outside as #asthma symptoms can be triggered by winter weather. #Staywellthiswâ€¦
Other	Tweets that do not fit in any of the above categories; recruitment for academic studies	
